# Influence of Preheating Temperature on Structural and Mechanical Properties of a Laser-Welded MMC Cobalt Based Coating Reinforced by TiC and PCD Particles

**DOI:** 10.3390/ma15041400

**Published:** 2022-02-14

**Authors:** Artur Czupryński, Mirosława Pawlyta

**Affiliations:** 1 Department of Welding Engineering, Faculty of Mechanical Engineering, Silesian University of Technology, Konarskiego 18A, 44-100 Gliwice, Poland; 2 Materials Research Laboratory, Silesian University of Technology, Konarskiego 18A, 44-100 Gliwice, Poland; miroslawa.pawlyta@polsl.pl

**Keywords:** LDMD, cladding, deposition, titanium carbide, synthetic polycrystalline diamond

## Abstract

This article presents research on the structural and mechanical properties of an innovative metal matrix composite (MMC) coating designed for use in conditions of intense metal-mineral abrasive wear. The layer, which is intended to protect the working surface of drilling tools used in the oil and natural gas extraction sector, was padded using the multi-run technique on a sheet made of AISI 4715 low-alloy structural steel by Laser Direct Metal Deposition (LDMD) using a high-power fiber laser (FL). An innovative cobalt alloy matrix powder with a ceramic reinforcement of crushed titanium carbide (TiC) and tungsten-coated synthetic polycrystalline diamond (PCD) was used as the surfacing material. The influence of the preheating temperature of the base material on the susceptibility to cracking and abrasive wear of the composite coating was assessed. The structural properties of the coating were characterized by using methods such as optical microscopy, scanning electron microscopy (SEM), energy dispersion spectroscopy (EDS), transmission electron microscopy (TEM) and X-ray diffraction analysis (XRD). The mechanical properties of the hardfaced coating were assessed on the basis of the results of a metal-mineral abrasive wear resistance test, hardness measurement, and the observation of the abrasion area with a scanning laser microscope. The results of laboratory tests showed a slight dissolution of the tungsten coating protecting the synthetic PCD particles and the transfer of its components into the metallic matrix of the composite. Moreover, it was proved that an increase in the preheating temperature of the base material prior to welding has a positive effect on reducing the susceptibility of the coating to cracking, reducing the porosity of the metal deposit and increasing the resistance to abrasive wear.

## 1. Introduction

Laser Direct Metal Deposition (LDMD) hardfacing—with direct feeding of metallic powder to the weld pool—is a very modern and advanced welding technology. The idea behind the process is to slightly melt the surface of the material with a heat source, a laser beam generated by a high-power laser, and simultaneously deliver a stream of metal powder to the weld pool through a nozzle. The additional material supplied from outside then becomes the main component of the produced coating, and as a result of the high temperature of the process, it melts with the substrate. The coatings produced using this method exhibit excellent metallurgical bonding to the substrate, low dilution (i.e., low mixing between the clad material and the substrate material, high density, little or no cracking and good mechanical properties. The LDMD technique and other types of laser surfacing can produce coatings from almost any metal material, both on new and regenerated elements, which are used in the following industries: aerospace, energy, petrochemical, automotive, and medical [1,2].

Recently, there has been a great deal of interest in the use of the laser cladding method to produce composite surface layers on the surface of components made of ferrous and nonferrous alloys [3,4]. Metal matrix composites (MMCs), due to their properties such as high hardness, excellent wear and corrosion resistance, are currently the most interesting—in terms of research and being the most desirable materials in industrial applications—group of additional materials for laser surfacing [5,6]. The combinations of metal matrix and reinforcing particles are very diverse, and the most popular are composites based on iron, nickel, cobalt, magnesium and aluminum alloys in combination with reinforcing particles such as tungsten carbide (WC) [7,8], silicon carbide (SiC) [9], boron carbide (B_4_C) [10] and titanium carbide (TiC) [11], the latter being used in this study.

Recently, many publications have referred to studies in which cobalt and its alloys were used as a matrix; however, such materials are seldom described as matrix materials in composite layers [12,13], and more often as homogeneous layers [14,15,16]. Cobalt–chromium alloys, called Stellites, which are characterized by high hardness, resistance to corrosive environments and, above all, high wear resistance (including metal–metal, metal–mineral and metal–liquid resistance) constitute a particularly important group of hardfacing materials used for the production of laser-clad surfaces on elements designed to work in conditions of high abrasive wear and extreme temperatures (up to 1050 °C), often combined with an aggressive working environment. In Stellites, the addition of chromium plays an important role—it is the main carbide-forming factor, and, as an alloying element in the matrix, it increases the strength and the resistance to corrosion, oxidation, and the effect of sulphur compounds. The very hard carbides present in the structure of Stellites create microscopic unevenness between sliding surfaces, which ensures high resistance to abrasive wear [17]. Despite this, efforts are still being made to improve surface properties and reduce the thickness of the overlay coating, resulting in an increase in the reliability and service life of machine and device parts and thus contributing to minimizing production losses [18,19,20]. For this reason, more innovative additional materials for surfacing and methods of their application are constantly being sought.

Composites based on cobalt alloys offer extremely broad prospects for research in this area. TiC particles are often used as reinforcements for composite layers in cobalt alloy matrices produced by laser surfacing processes [21,22,23]. They are used as both volumetric and surface reinforcement. Shasha et al. [13] investigated the mechanical properties of a coating based on a cobalt alloy reinforced in situ with TiC, TiB_2_, Cr_5_Si_3_, WB, SiC, Co3Ti and NiC particles produced on the surface of a TA15 titanium alloy using a cross-flow CO_2_ laser. The results showed that the diversified and numerous interstitial phases dispersed between fine dendritic structures in a matrix mainly composed of γ-Co, α-Ti solid solution improved by more than threefold the hardness of the coating compared to the substrate. Compared to the titanium alloy, the abrasion resistance of the coating was significantly improved, and the wear rate of the coating was about 1/12 of the titanium alloy. The abrasive wear mechanism of the hardfaced coating was mixed.

TiC-Co LMD (Laser Metal Deposition) composite coatings produced on a ductile iron substrate were investigated by Tong et al. [21]. The results showed that an increase in the size of the primary or secondary dendrite can be inhibited by the separation of TiC after its dissolution in the molten metal pool. The hardness on the surface of the coating gradually increased (to 1246.6 HV0.2) with a decrease in laser power or an increase in the scanning rate. Attention was paid to the importance of the size and shape of the hard phase particles and the type of matrix material. Unfortunately, the paper did not present any tribological tests. Zahang [22] produces a composite TiC-Co layer laser welded onto a substrate made of 2Cr13 steel. The layer was intended for preventive protection of the working surface of machine parts and power devices exposed to abrasive wear and impact load. The structure of the shell was highly diversified and consisted of several sublayers. As a result of the impact of the laser beam, the surface material became harder, while fusion and a transition zone occurred further within the coating. The coating structure consisted of supersaturated cobalt dendrites with dispersed TiC particles. The test results showed that, for steel, it was possible to strengthen the welded material by simply hardening it. However, in the case of superalloys, as a result of mutual diffusion and the mixing of the alloy and substrate components, the chemical composition and structure of the top coat changed, which was undesirable. Moreover, it was found that a higher amount of the hard TiC phase promoted the growth of the metal matrix grain. The abrasive wear test showed relatively good metal-to-metal wear resistance.

Many more pieces of experimental data have been published on laser-welded composite layers employing a matrix of cobalt alloys reinforced with WC [24,25,26,27]. Most of the research concerns the dissolution of WC in the matrix and its influence on the final properties of the coatings. According to Zanzarin et al. [25], the dissolution of WC during the laser surfacing process increased with an increase in the amount of heat supplied, an increase in the temperature of the preheating of the base material or a decrease in the tungsten and carbon content in the matrix. Dissolving WC can increase the susceptibility to cracking and reduce the resistance to abrasive wear. An interesting alternative to WC, due to its lower density and much higher values of Young’s modulus, compressive strength, flexural strength, fracture toughness and hardness, is a synthetic polycrystalline diamond (PCD). However, the decomposition temperature of pure synthetic PCD (1450 °C) is much lower than the decomposition temperature of tungsten carbide (2870 °C). There are no data in the scientific literature on the use of synthetic PCD as a matrix reinforcement of MMC composite layers produced by LMD.

**Figure 1:**
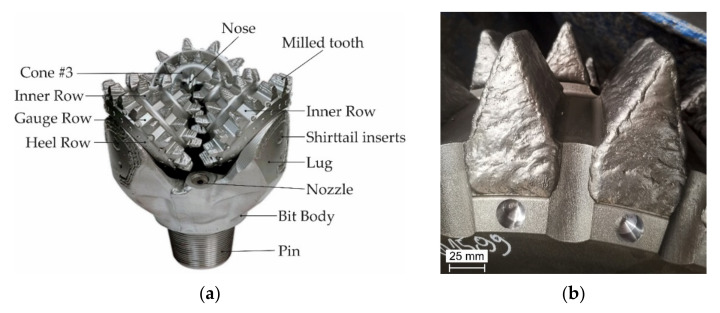
Three-cone bit with milled teeth: (**a**) general view of the drill bit with milled teeth, (**b**) working surface of the teeth after oxyacetylene hardfacing.

The aim of the study was to determine the effect of the preheating temperature of the base material on the structure, susceptibility to cracking, and abrasion resistance of a laser-welded coating consisting of composite powder in a cobalt alloy matrix. A novelty in the presented results is the development of an innovative composition of the matrix reinforcement consisting of a composite containing super-hard phases in the form of ceramic particles from finely crushed TiC and spherical particles made of synthetic PCD, the latter being protected against thermal decomposition with a tungsten coating. Our results showed that there is a possibility of producing a composite laser-welded coating using the ceramic reinforcement—the cobalt matrix phase system, the microstructure and abrasive properties of which are appropriate to protect the working surface three-cone toothed bits [28]. Currently, laser surfacing of the working surface of drilling cutter teeth is not used under production conditions. Typically, these elements are protected against wear by hardfacing using the oxyacetylene welding (OAW) method with a composite stick (tubular electrode) with a powder core, Figure 1b. The implementation of the LMD surfacing technology in place of the gas surfacing technique used up to now for this industrial application may contribute to an improvement in the quality of surface layers by minimizing noncompliance, stresses, and welding deformations, as well as the share of base metal dilution in the weld, even to levels below 3%. In addition, it will allow for obtaining the required chemical composition of the padding weld to be already obtained in the first layer, contributing to greater production efficiency resulting from the automation and robotization of the padding process, and allowing for the finishing time of the padded surface to be shortened.

## 2. Materials and Methods

### 2.1. Materials

**Figure 2:**
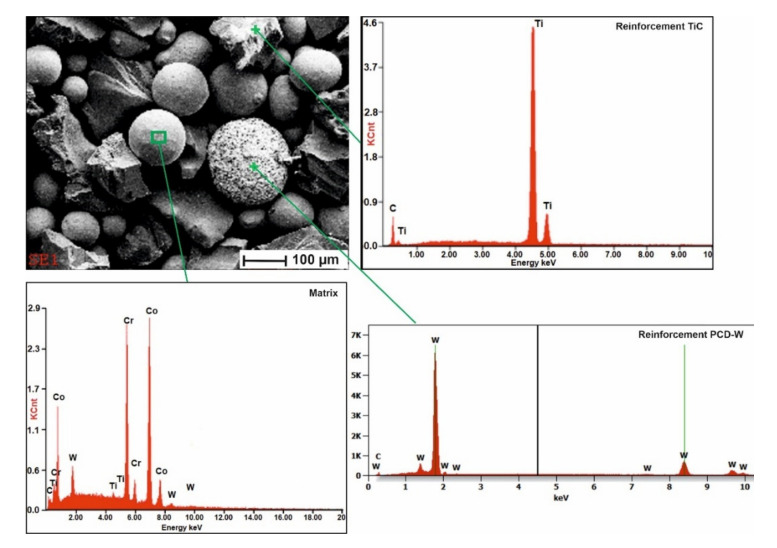
SEM image of the composite powder particle morphology and EDS spectra obtained for selected grains.

The material used for surfacing was an innovative metallic powder of the Co3 alloy group (Höganäs AB, Höganäs, Sweden) according to EN 147000 [29], characterized by an increased weight fraction of carbon (up to 3%) and tungsten (up to 14%). The metal powder that constituted the composite matrix material was mechanically mixed in a Turbula T2F laboratory turbulent mixer (Glen Mills Inc., Clifton, NJ, USA). The powder consisted of a hard reinforcing phase composed of crushed sharp-edged titanium carbide TiC and spherical synthetic polycrystalline diamond PCD (Harmony Industry Diamond, Zhengzhou, China) with a tungsten coating. The proportion of the reinforcing phase components to the metal matrix components was 60% to 40% by weight with the hard phase content composed of 90% TiC with a particle size ranging from 140 to 250 µm and 10% PCD with a particle size of 180 µm, Figure 2. The chemical composition of the composite powder, claimed by patent P.435997, is presented in Table 1. The base material was a low alloy structural steel of AISI 4715 (Table 2).

### 2.2. Laser Processing

**Figure 3:**
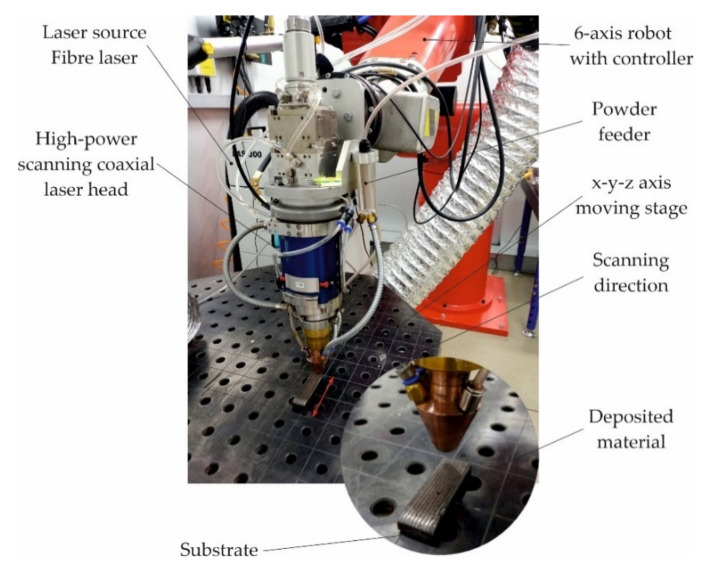
View of the test stand used for robotic surfacing with the LDMD.

The laser powder surfacing process was carried out using the LDMD technique—with direct feeding of the composite powder to the weld pool—on a robotic stand equipped with a modern surfacing system using a YLS-4000 ytterbium fibere laser system (IPG Photonics Corporation, Oxford, MA, USA) with a wavelength of λ = 1070 nm and a maximum laser beam power of 4000 W mounted on a REIS RV30-26 six-axis robot (Reis Robotics, Obernburg am Main, Germany), Figure 3.

**Figure 4:**
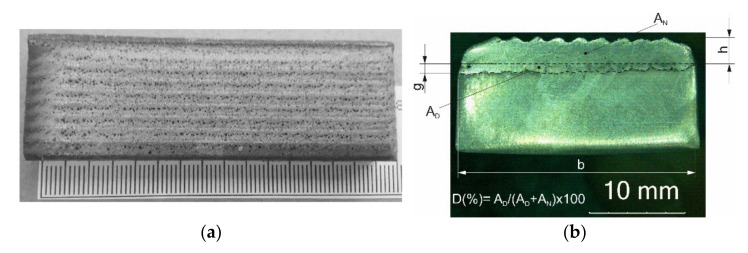
LDMD coating with composite powder: (**a**) a view from the face of the padding welds, (**b**) a cross section of the coating.

The diameter of the laser spot at the focal plane, 20 mm from the nozzle tip, measured with a UFF100 Laserscope (Prometec GmbH, Aachen, Germany), was 5 mm, with energy distribution in the TEM01* beam. The powder cladding set-up consisted of a computer-controlled powder feeding system and a coaxial cladding nozzle integrated with a Computerized Numerical Control (CNC) five-axis gantry. Coaxial powder injection was realized by using nozzle gas, carrier gas and shielding gas—Argon 5.0 (99.999%). Cladding was performed at the following gas flow rates: nozzle gas (Ar) = 15 L/min, carrier gas (Ar) = 2.8 L/min and shielding gas (Ar) = 12 L/min. In order to determine the range of optimum surfacing parameters, a series of single-run padding welds was carried out with laser power values of 1400, 1600, 1800, 2000 and 2200 W with a surface speed from 4 to 12 mm/s and a powder feed rate from 15 to 30 g/min. The optimum parameters for the surfacing of the composite coating (Table 3) were defined as the parameters that ensured a uniform distribution of the powder across the entire surface of the liquid metal in the weld pool, the correct depth of fusion g < 2 mm, a layer height h < 5 mm and a percentage of dilution of the base metal in the surface layer D < 5% (Figure 4).

Test samples were produced with dimensions of 75 × 25 × 10 mm—in a series of three, welded either without or with preheating of the substrate using an oxyacetylene torch to temperatures of 100, 200 or 300 °C, and the samples were marked with the symbols T0 (no heating), T100, T200 or T300, respectively.

### 2.3. Methodology of Research

In order to assess the quality of the surfacing welds and to determine the number of cracks in the surfacing layers at different preheating temperatures of the substrate, non-destructive testing i.e., visual tests (VT) and penetration tests (PT) were carried out. The structural and mechanical properties were determined on the basis of the analysis of the results of macro- and microscopic metallographic tests, chemical composition, X-ray diffraction, hardness, and porosity measurements as well as metal-mineral abrasive wear resistance tests.

#### 2.3.1. NDT Tests

Visual tests (VT) and penetrant tests were carried out in accordance with the guidelines contained in the relevant standards, i.e., ISO 17637 [30] and ISO 3452-2 [31]. VT consisted of determining, using the naked eye and a digital pen microscope, the location and assessment of the surface quality features of the coating, such as cracks, porosity, spikes, undercut, imperfect shapes, and dimension. Before starting the actual test, the tested surface was prepared by thoroughly cleaning it of all impurities and drying it. The system of color preparations (system design type II, sensitivity 2) PT ISO 3452-2 II Cd-2 and EN 571-1 was used for penetration tests.

#### 2.3.2. Metallographic Examination and X-ray Diffraction Analysis

Microscopic examinations were performed on metallographic specimens prepared by standard methods. The etching reagent was a mixture of concentrated hydrochloric acid and nitric acid in a volume ratio of 3:1, the so-called ‘aqua regia’, and the digestion time of the sample was experimentally selected. The observation and saving of macro- and microstructure images were performed using an Olympus SZX9 stereoscopic microscope (Olympus Corporation, Tokyo, Japan) equipped with a Moticam 5.0+ digital camera (Motic (Xiamen) Electric Group Co Ltd., Xiamen, China) and Motic Images Plus 3.0 software (Motic (Xiamen) Electric Group Co Ltd., Xiamen, China). SEM studies were performed on a Zeiss Supra 35 microscope (Carl Zeiss SMT, Oberkochen, Germany) equipped with an EDS spectrometer for chemical composition analysis. For transmission electron microscopy (TEM) observations, samples were prepared employing the Focused Ion Beam (FIB) technique using an SEM/Ga-FIB Helios NanoLabTM 600i microscope (FEI Company, Hillsboro, OR, USA). TEM investigations were performed using an S/TEM Titan 80-300 microscope (FEI Company, Hillsboro, OR, USA) equipped with a Cetcor Cs probe corrector (CEOS, Heidelberg, Germany) and an EDS and EELS spectrometer for chemical composition analysis. Crystal Maker (version 10.4.1) and Single Crystal software (CrystalMaker Software Limited, Oxfordshire, UK) were used to simulate the crystal structure and diffraction patterns. The phase composition of the hardfaced coating was determined by X-ray diffraction tests performed on at Panalytical X’Pert Pro MPD diffractometer (Malvern Panalytical Ltd., Malvern, UK), using filtered radiation (Kβ Fe filter) from cobalt anode lamps (λKα = 0.179 nm). The diffractogram was recorded in Bragg—Brentano geometry, using a PIXcel 3D detector on the axis of the diffracted beam over an angle range of 20–110 [2θ] (step = 0.05°, count time per step = 100 s). The diffractograms obtained were analyzed using dedicated Panalytical High Score Plus software (Malvern Panalytical Ltd., Malvern, UK) together with the PAN-ICSD structural database. Quantitative phase analysis of the X-rays was performed using the Rietveld method.

#### 2.3.3. Density Measurement and Examination of the Porosity of the Coating

The density of the top coat was measured according to ASTM D792 [32] using a Radwag AS 220.R2 analytical laboratory balance (Radwag, Warsaw, Poland) with the Archimedes method of density measurement.

The porosity of the surface coating was assessed using a µCT Nanotom 180N microtomography device (Ge Sensing & Inspection Technologies GmbH, Wunstorf, Germany) equipped with an X-ray tube with a maximum voltage of 180 kV. Tomographic images were recorded using a Hamamastu 2300 × 2300 pixel decoder (Hamamatsu Photonics K.K., Hamamatsu, Japan). The virtual reconstruction of the shell structure was mapped using proprietary GE datosX ver.2.1.00 software (GE Sensing & Inspection Technologies GmbH, Hürth, German). All tomographic images were taken at a source voltage of 140 kV and 200 µA with a 360° rotation of the element in 2400 steps. The exposure time was set at 500 ms, the frame averaging at 3 and the image skipping at 1, while the scanning time of a single element t = 80 min. Porosity analysis was performed using the MyVGL program (Volume Graphics GmbH, Heidelberg, Germany).

#### 2.3.4. Hardness Measurements

The hardness on the surface of all coatings (samples symbols T0, T100, T200 and T300) was measured using the Rockwell C method, according to the procedure described in ISO 6508-1 [33]. The tests were carried out using a Sunpoc Super Rockwell SHRS-450M hardness tester (Guizhou Sunpoc Tech Industry Co., Ltd., Guizhou, China) on the minimally sanded outer surface of the hardfacing coating, at five measuring points located on the axis of the sample with a 10 mm space between them. Hardness measurements in the cross section of the overlay coating were performed using the Vickers method, according to the guidelines contained in the ISO 6507-1 standard [34]. The measurement was carried out on metallographic specimens (T0, T100, T200 and T300) at 10 measurement points, separately for the hard phase particles and the metal matrix, and the hardness was determined on the HV 0.05 scale. The tests were performed using a hardness tester with an automatic measuring track and a Future-Tech FM-ARS 9000 image analysis system (Future-Tech Corp., Kawasaki, Japan).

#### 2.3.5. Abrasive Wear Test

**Figure 5:**
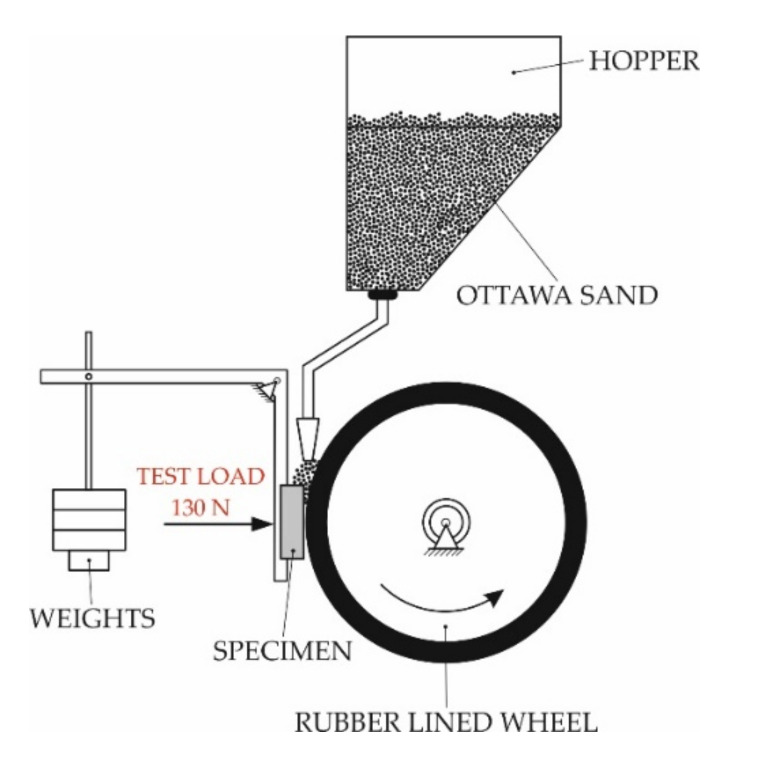
Schematic diagram of the stand for testing resistance to metal-mineral abrasive wear according to the ASTM G65 standard.

The metal-mineral wear resistance of the surface layer was tested according to the ASTM G 65-00 standard, Procedure A [35]. The abrasive wear process was tested with the use of a ’rubber wheel’ type device (Figure 5).

The surface coating abrasion test, without heating and with preheating of the base material to temperatures of 100, 200 and 300 °C, was carried out for four series of two samples for each initial temperature of the base material. During the test, which lasted about 30 min, the friction wheel made 6000 revolutions against the test material with a force of 130 N and a flow rate of the abrasive material (A. F. S. Testing Sand 50–70 mesh) of 335 g/min.

Before and after the abrasion test, the samples were weighed on a laboratory balance with an accuracy of 0.0001 g. The average coating density was determined using a laboratory balance, on the basis of three density measurements of samples taken from the tested materials and weighed at room temperature in air and liquid. Using the measured average density of the hardfaced coating and the average mass loss of the sample after the abrasion test, the volumetric mass loss was calculated according to Equation (1):

(1) $$\mathrm{Volume}\;\mathrm{loss}\;\left[{\mathrm{mm}}^{3}\right]=\frac{\mathrm{mass}\;\mathrm{loss}\;\left[g\right]\;}{\mathrm{density}\;\left[\frac{g}{{\mathrm{cm}}^{3}}\right]\;\;}\times 1000$$

The surface abrasion area of the coating after the ASTM G65 test was observed using a 3D LEXT™ OLS5100 laser scanning microscope (Olympus Corporation, Tokyo, Japan), allowing a very precise measurement of the shape of the abrasion profile and surface roughness.

Furthermore, based on planimetric tests carried out on macroscopic samples (Figure 4b), the share of base metal dilution substrate material in the composite layer was estimated according to Equation (2):

(2) $$D=\frac{A_{N}}{A_{N}+A_{D}}\times 100\%$$

where A_N_ is the area of the fusion of the layer into the base material, and A_D_ is the rim area of the weld overlay.

## 3. Results and Discussion

### 3.1. Non-Destructive Testing Results

**Figure 6:**
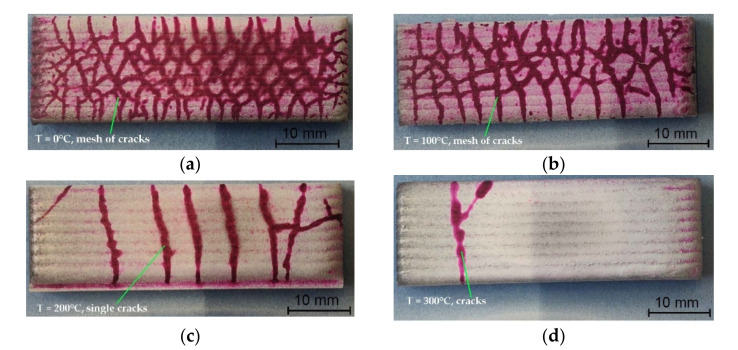
View of the coating laser-welded with composite powder after penetration tests (PT): (**a**) surfacing without heating, surfacing with preheating of the base material to temperatures of (**b**) 100 °C, (**c**) 200 °C, (**d**) 300 °C.

Non-destructive testing of laser-welded composite layers of cobalt Co3 powder containing of TiC and PCD ceramic particles on the substrate of low-alloy AISI 4715 structural steel allowed the type, location and size of surface imperfections to be determined. The results of penetration tests of surface coatings without heating or with preheating of the base material to temperatures of 100, 200 or 300 °C are shown in Figure 6. 

In terms of the quality of workmanship, the coatings were characterized by a high regularity of the outer plane and the symmetry of the successive overlapping, weld beads. VT and PT of the surface of the layers showed only the presence of radial cracks (1051) and transverse cracks (1021) [36]. The lack of preheating of the substrate before surfacing resulted in the appearance of a dense network of cracks, Figure 6a. Increasing the preheating temperature to 100 °C caused the crack mesh density to decrease, and in the temperature range from 200 to 300 °C, a significant reduction was observed in the number of cracks in the layer. Only single cracks were detected spreading throughout the coating width in a line perpendicular to the welding direction, Figure 6c,d. A higher content of matrix alloying additives and a higher proportion of hard phase ceramic particles reinforcing the composite contributed to an increase in abrasion resistance, but at the same time increased the tendency for surface cracks to form in the padding weld. Cracks appeared during cooling due to the difference in the thermal expansion coefficients of the base material and the padding weld material [37]. The cracks most often penetrated deep into the base material and in no way weakened the adhesion of the surface coating.

**Figure 7:**
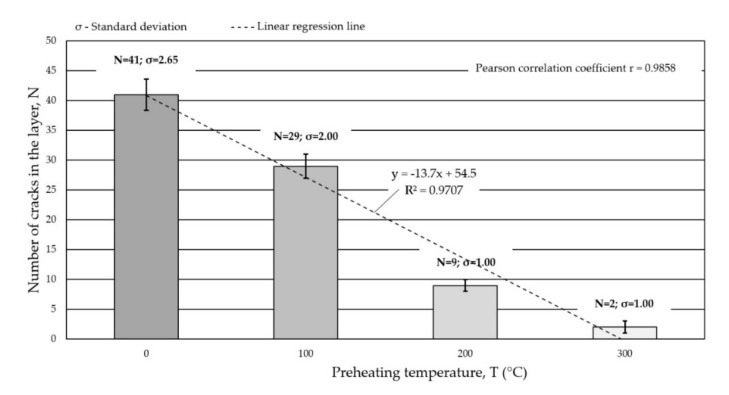
Influence of the preheating temperature of the base material before surfacing on the number of cracks in the composite layer.

According to [38], in wear resistant MMC layers, cracks are acceptable if they appear across the seams and the distance between them is not less than 20–30 mm. In the case of the composite tested, this condition was met for the preheating temperature of 300 °C. To obtain a crack-free surfaced coating, it was necessary to select the preheating temperature and control the inter-pass temperature. Preheating the material before surfacing reduces shrinkage stress [39] and the hardness in the heat-affected zone [40], while also reducing the risk of hydrogen cracks [41]. In the analyzed case, the factors that influenced the necessity to use preheating were the high carbon equivalent value, CEV = 0.66% and the high-power density of the heat source, which translated into low linear welding energy. For a given configuration of the base material and filer metal, the preheating temperature should exceed 300 °C and should be maintained throughout the welding cycle. The size and thickness of the welded material are also important in this case. Elements with larger dimensions, such as toothed cones, should be heated more (even up to 500–700 °C), bearing in mind that too high a preheating temperature can, however, contribute to the thermal decomposition of the carbide phase [42]. The effect of the preheating temperature of the base material before surfacing on the number of cracks in the composite layer is shown in Figure 7.

### 3.2. Metallographic Test Results and Results of the XRD Analysis

**Figure 8:**
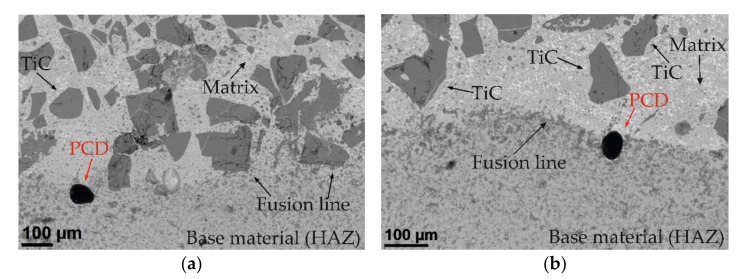
Area of the composite shell fusion line. SEM images (BSE detector): (**a**) left side of the coating section and (**b**) right side of the coating section.

**Figure 9:**
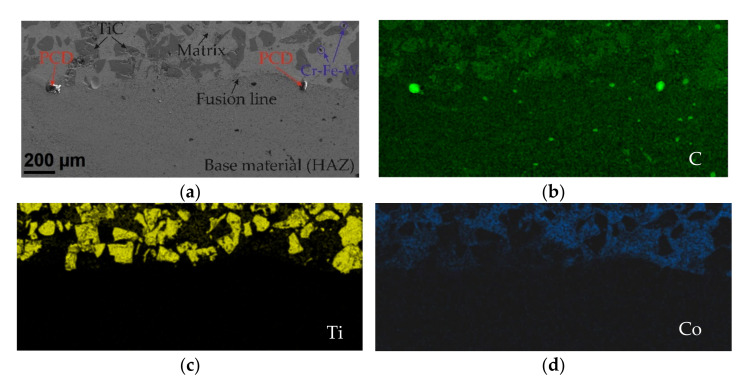
Area of the fusion line of the composite shell. (**a**) SEM image (SE detector). EDS maps of of element distribution, (**b**) carbon—green, (**c**) titanium—yellow, (**d**) cobalt—blue.

The results of microscopic metallographic observations of the composite coating allowed the matrix structure as well as the type, distribution and dimensions of the ceramic reinforcement to be determined. SEM observations were carried out at 80, 500 and 1500 times magnification. Secondary Electron (SE) as well as Back Scattered Electron (BSE) detectors were used for image acquisition, the latter to more clearly show the chemical contrast, ensuring the highest image quality. For detailed structural analysis of the areas containing carbide reinforcement particles, a transmission electron microscope (TEM) was used to determine the grain size, structural defects and cracks in the TiC and PCD particles. The results of the microscopic observations are shown in Figure 8 and Figure 9.

Scanning microscopy studies showed that the metal matrix of the composite coating was densely and evenly filled with a large number of inclusions of ceramic TiC particles and single particles of synthetic PCD. Figure 8 shows the fusion zone of the coating deposited against the base material. Two areas separated by a fusion line are visible, differing in morphology and chemical composition. In the surfacing layer, geometrically irregular TiC particles with grain sizes often exceeding 100 µm can be observed. TiC is known to have good mechanical properties and is a frequently used ceramic additive for composite binders (MMCs) designed to protect elements exposed to high abrasive wear in combination with impact stress [43]. However, like most ceramics, titanium carbide is brittle and, due to the reduction of plasticity by strong bonds, has a tendency to undergo catastrophic fracture [44,45]. According to Sun et al. [46], cracking occurs during plasma surfacing (PPTAW), in the first phase of cooling of the liquid metal in the weld pool, due to the concentration of tensile stresses in carbide defects. The tests conducted showed that, during the LDMD method, the tendency of the TiC particles to fracture clearly decreased with an increasing preheating temperature of the base material.

**Figure 10:**
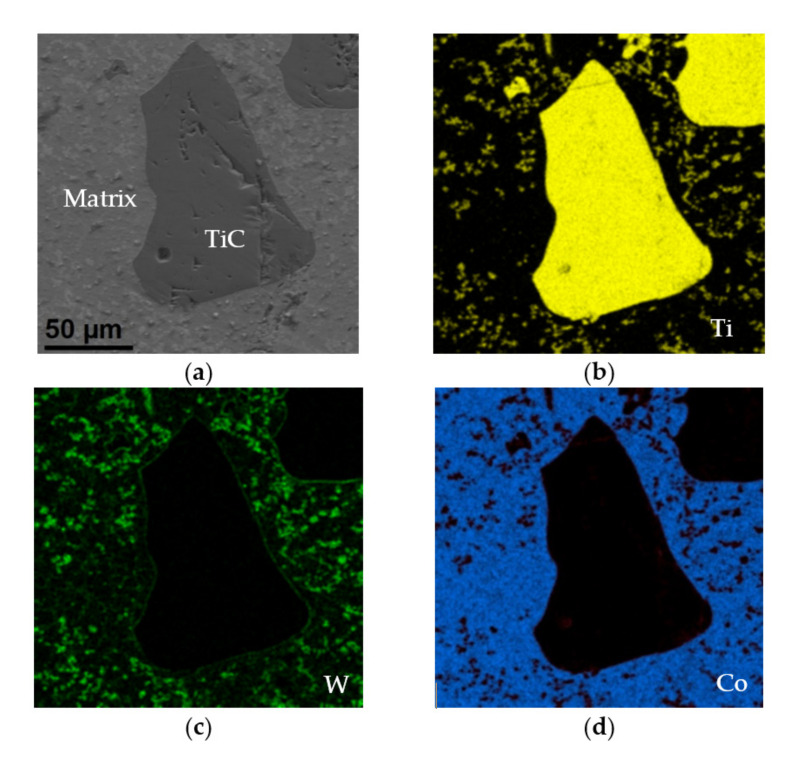
Titanium carbide particle: (**a**) SEM image (SE detector) and EDS maps of element distribution, (**b**) titanium—yellow, (**c**) tungsten—green, (**d**) cobalt—blue.

Figure 10 shows a single grain of titanium carbide with a size of approximately 200 μm. In the matrix surrounding it, much finer grains (of a size of a few micrometers) enriched with tungsten and titanium are noticeable.

**Figure 11:**
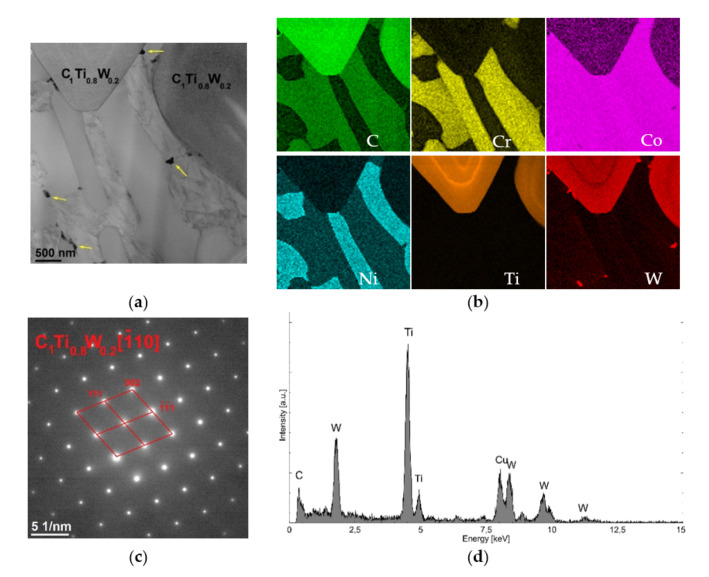
Structure of the area containing C1Ti0.8W0.2 carbides: (**a**) TEM image, (**b**) maps of C, Cr, Co, Ni, Ti and W distribution (C and Ti determined by EELS, other by EDS), (**c**) SAED electron diffraction of C1Ti0.8W0.2 in the [–110] direction and (**d**) EDS spectrum of C1Ti0.8W0.2 carbide.

In BSE images (Figure 8), they are visible as brighter spots. To characterize them in detail, TEM studies were performed. Figure 11a shows a smaller area (approximately 4 μm × 4 μm) taken from the surroundings of the TiC.

It included two fragments of grains containing carbon, titanium and tungsten, and a cobalt matrix in which the presence of Cr, C, Ni, Ti and W (Figure 11b) was confirmed. Due to the use of the EELS spectrometer, it is possible to clearly confirm the presence of C in the analyzed grains containing Ti and W. The EDS analysis (Figure 11d) showed that the atomic share of Ti and W was 80% and 20%, respectively (ESD does not allow for a reliable quantitative analysis of light elements; therefore, the C content is not included here). The presence of Cu in the spectrum resulted from its presence in the structure of the microscope and can be ignored. Based on Selected Area Electron Diffraction (SAED) (Figure 11c), the analyzed grains were identified as the C1Ti0.8W0.2 phase (98-007-7553, cubic structure, space group Fm-3m).

**Figure 12:**
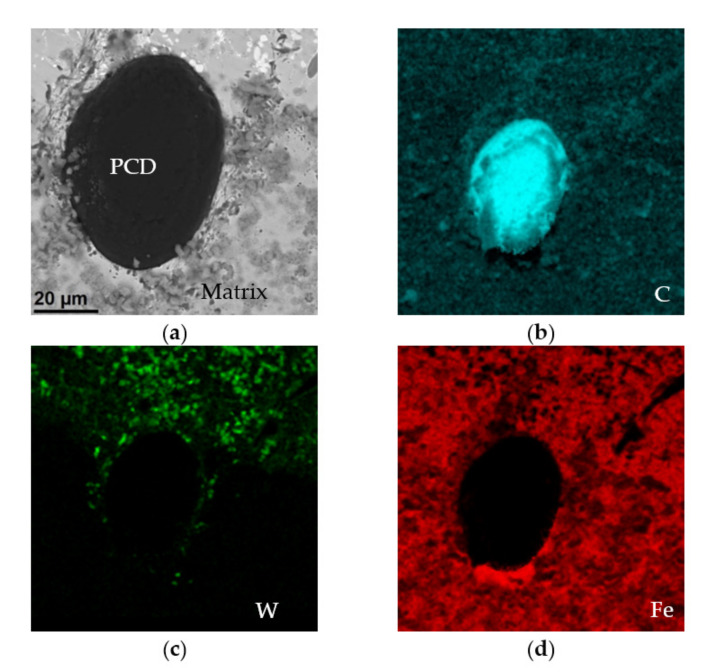
PCD particle embedded in the substrate material: (**a**) SEM image (BSE detector) and element distribution maps: (**b**) carbon—blue, (**c**) tungsten—green, (**d**) iron—red.

In addition, two large grains of synthetic PCD were observed in the fusion line, Figure 9. The particles, which were partially fused into the base material, had an oval shape and sizes of up to several dozen micrometers. Single grains of sintered diamond were also observed in the central part of the cross section of the surface coating, as well as in the vicinity of the padding weld. The results of the analysis of the chemical composition of the synthetic PCD grain embedded in the substrate material and the matrix fragment surrounding it are presented in Figure 12.

**Figure 13:**
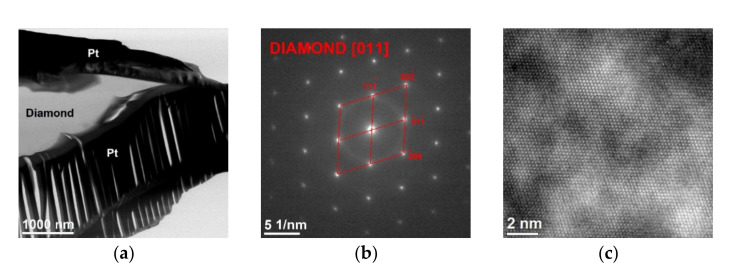
Synthetic PCD: (**a**) TEM image—BF, (**b**) SAED diffraction of the PCD particle towards [011], (**c**) HRTEM image.

In order to fully characterize the grain structure of the PCD particles, TEM observations were performed. It is worth noting that, because of the large differences in the hardness of the components of the tested material (diamond-matrix), making lamellas using the FIB technique turned out to be extremely difficult. The obtained lamellas had a heterogeneous thickness, contained significant amounts of Pt deposited for protective purposes and were subject to strong deformation as a result of stresses. Figure 13 shows a TEM image of a lamella with a fragment of a PCD particle.

Electron diffraction has a selected area aperture (Figure 13b) of a diamond structure (96-901-1576 cubic structure, Fd-3m space group), while high resolution HRTEM imaging of its crystal showed a non-defective structure (Figure 13c).

It was noted that some of the tungsten from the protective coating that covered the PCD particles went into solution, Figure 12a,c. The rest of the element chemically reacted with the carbon contained in the diamond to form a thin WC coating. This coating had good thermal stability, ensured the cohesion of the particle, increased its strength, and also improved the thermal conductivity of the alloy, which could indirectly increase its resistance to abrasive wear.

**Figure 14:**
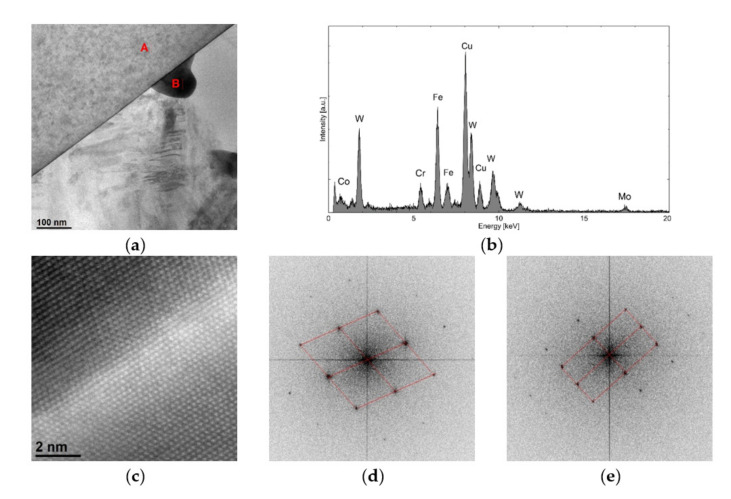
Precipitate of Cr0.4Fe0.475W0.125 on the boundary of C1Ti0.8W0.2 carbide: (**a**) TEM image, (**b**) EDS analysis of the precipitate of Cr0.4Fe0.475W0.125 from the area marked as B, (**c**) STEM– HAADF image of the boundary between the C1Ti0.8W0.2 carbide and the precipitate of Cr0.4Fe0.475W0.125, (**d**) Fourier transformation of the area denoted as A (identified as C1Ti0.8W0.2 in the [–110] direction), (**e**) Fourier transformation of the area denoted as B (identified as Cr0.4Fe0.475W0.125 in the [–110] direction).

In Figure 11a, there are several small irregular particles that contain tungsten. The size of the particles did not exceed several dozen nanometers. Precipitates of this type were only located in the area of the fusion line, where the iron content in the matrix was high. An example of such a precipitate located on the C1Ti0.8W0.2 carbide boundary (marked A) is shown in Figure 14a.

The precipitate (marked B) contained 46% Fe, 22% W, 12% Cr, 16% Co and 4% Mo (Figure 14b). Based on the STEM-HAADF image obtained and the Fourier transformation (Figure 14c–e), the analyzed precipitate was identified as the Cr0.4Fe0.475W0.125 phase (98-062-6001, cubic structure, space group I-43m).

The properties of cobalt alloys are largely due to their crystallographic structures. In the developed powder, alloying elements such as Fe, Ni and C stabilize the cubic structure A1 of cobalt, which below a temperature of 417 °C is transformed into crystals of the densely packed hexagonal A3 lattice stabilized by Cr, W and Mo. At ambient temperature, a metastable regular flat-centered phase is often present in cobalt alloys instead of the hexagonal phase. This phase, defined as cobalt (alloy) austenite, is a solid solution of Cr, Ni, Fe, W, Mo or Mn in cobalt [42].

The chemical composition of the coating, especially in the area of the fusion line, differed from the chemical composition of the filler metal used for surfacing due to the proportion of the substrate in the layer. The powder used for the surfacing contained up to 5% Fe, while in the layer an increased presence of this element was found. The matrix microstructure of the composite was chemically heterogeneous. The dendritic area was cobalt austenite strengthened by a solution of elements such as chromium, tungsten or molybdenum. Interdendritic eutectics were rich in chromium, tungsten, silicon and carbides. The Cr alloy was designed to provide corrosion resistance and strengthen the solid solution by creating M_7_C_3_ and M_23_C_6_ carbides. Tungsten reached a content of 22% in some areas of the coating, contributing to the strengthening of the solid solution and favoring the formation of MC and M6C carbides and intermetallic phases [47].

**Figure 15:**
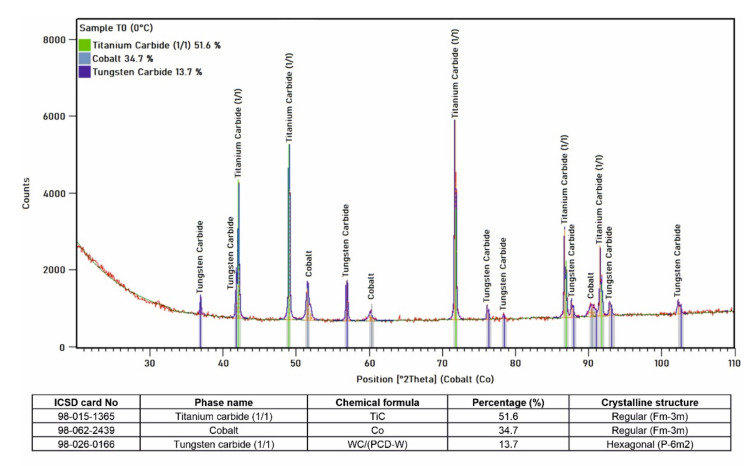
X-ray diffraction pattern of the composite coating welded by LDMD with marked reference lines of the identified crystalline phases and the results of X-ray qualitative phase analysis.

X-ray diffraction phase and quantification analyses were performed to identify the phases present in the layer. The diffractogram and the result of the X-ray qualitative phase analysis are presented in Figure 15. The analysis showed less than 35% of γ-Co (cobalt austenite) and over 51% of regular carbide with the TiC structure, as well as 14% of hexagonal carbide with a WC structure. The indication of WC probably comes from carbide phases formed on the surface of the synthetic PCD.

### 3.3. Hardness Measurements’ Test Results

**Figure 16:**
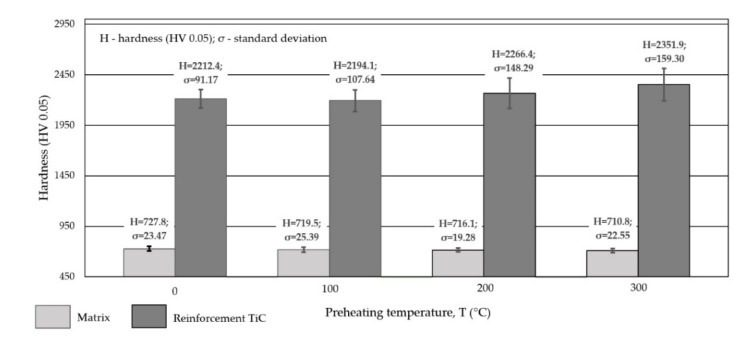
HV hardness measurement results on the cross-section of the coating laser-padded with Co3+TiC+PCD-W composite powder on AISI 4715 low-alloy structural steel.

Measurements of the hardness of the outer surface and the cross-section of the coating are presented in Table 4 and Figure 16.

It was assumed that, as the amount of heat supplied to the substrate material increased, the dissolution of the PCD particles would increase, which would affect the hardness of the coating in two ways. First, it was expected that, as PCD particle dissolution increased, the hardness would decrease as the volume fraction of the ceramic phase decreased. At the same time, it was expected that the average microhardness of the alloy matrix would increase as a result of the dissolution of tungsten and carbon. Furthermore, it was taken into account that the hardness of the coating would also be influenced by the dilution ratio of the substrate due to the dissolution of iron and carbon from the substrate. The results of the tests presented in Table 4 show that, even in the case of a large share of the base material in the padding weld, the hardness measurements on the surface of the layer show only a slight downward trend. The share of base material did not affect the final microhardness of the alloy matrix (Figure 16), and thus did not affect the overall hardness of the coating.

In the Co-Cr-W-Ni-Fe-Mn-Mo-C alloy, there was no intensive dissolution of the tungsten coating protecting the PCD particle, and tungsten and carbon were already present in the matrix. The transfer of these two elements to the weld metal slightly enriched the alloy matrix with tungsten and carbon and had no significant effect on the microhardness of the cross section of the composite coating.

The hardness of the surface layer tested on the outer surface, depending on the preheating temperature of the substrate material, varied in a range from 58.7 to 60.9 HRC, Table 4. An increase in the preheating temperature of the substrate material by 300 °C in relation to the material not heated before surfacing resulted in a slight decrease in hardness, which was slightly more than 2 HRC. However, the measurements carried out on the cross section of the top coat showed that, depending on the preheating temperature, the average microhardness of the areas between the dendritic areas of the alloy on the cobalt alloy matrix ranged from slightly more than 710 HV0.05 (60.5 HRC) to almost 728 HV0.05 (61.3 HRC). 

With an increase in the preheating temperature of the base material, a slight decrease in the average hardness of the composite matrix was observed. The reason for this may be the partial dissolution of the PCD particles and the transfer of tungsten and carbon into the metallic matrix. This explanation is confirmed by the research conducted by Zanzarin et al. [25] and Janicki [27]. Moreover, a slight increase in the microhardness value was found as the measuring point moved away from the fusion line toward the coating surface. This trend may have been influenced by the dissolution of the iron and carbon composite from the substrate into the matrix. The microstructure of the coating in the area adjacent to the heat affected zone differed from that of the padding weld subsurface coating because of the method of crystallization of the liquid metal. Higher hardness may have also resulted from a large number of hard phase particles at the surface of the padding weld. The average hardness of the TiC particles that made up the reinforcement of the matrix was about 2256 HV0.05, and it should be noted that the hardness of the synthetic PCD particles could not be measured. 

### 3.4. The Results of Density Measurement and Testing the Coating Porosity 

**Figure 17:**
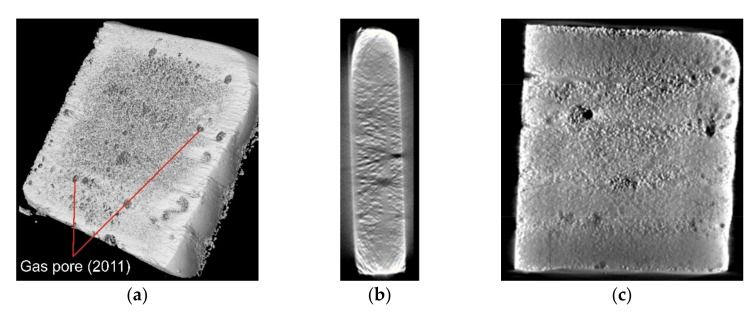
View of the µCT structure of the composite coating welded using the LDMD method, sample T300: (**a**) view of the longitudinal section from the side of the padding weld face, (**b**) view of the coating cross-section surface, (**c**) view of the longitudinal section surface from the base material side.

On the basis of the measurements of the mass of the samples taken from the composite layers, calculations were carried out regarding the variation of the specific density of the composite and the degree of its porosity with regard to the preheating temperature of the base material. The results of the measurements and calculations for three samples in each series are presented in Table 5. Examples of images of the structure of the composite coating obtained as a result of the µCT analysis are shown in Figure 17.

The composite layer tested was not free of internal porosity. The overall porosity of the coating decreased from more than 10% to less than 3% as the substrate preheat temperature increased. The lower amount of heat supplied to the welded material and the narrow fusion zone—although advantageous for technological and aesthetic reasons—may have impeded the escape of metal vapors from the steam channel and thus promoted the formation of bubbles and porosity in the padding weld. Preheating the base material before padding reduces the rate of crystallization of liquid metal in the weld pool and promotes the desorption of accumulated gases [26,48].

### 3.5. Abrasive Wear Test Results

**Figure 18:**
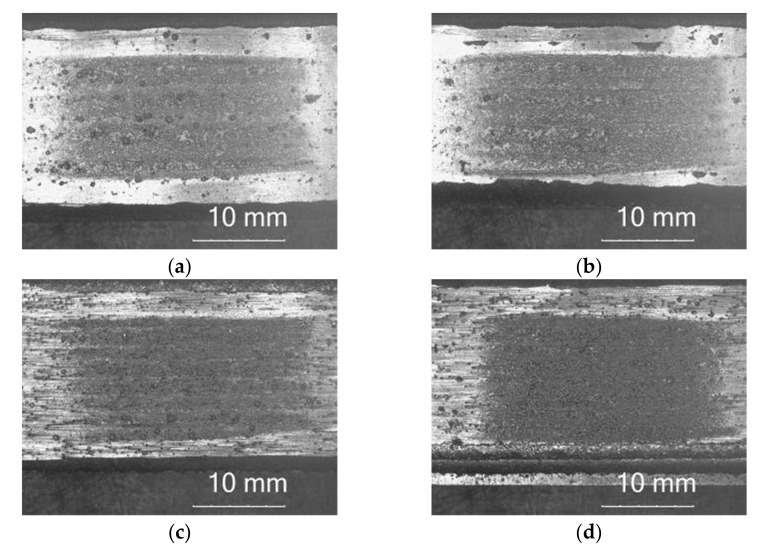
View of the surface of the abrasion area after the metal-mineral abrasion test of a composite coating welded by LDMD: (**a**) without heating the substrate and with heating to a temperature of: (**b**) T = 100 °C, (**c**) T = 200 °C, (**d**) T = 300 °C.

**Figure 19:**
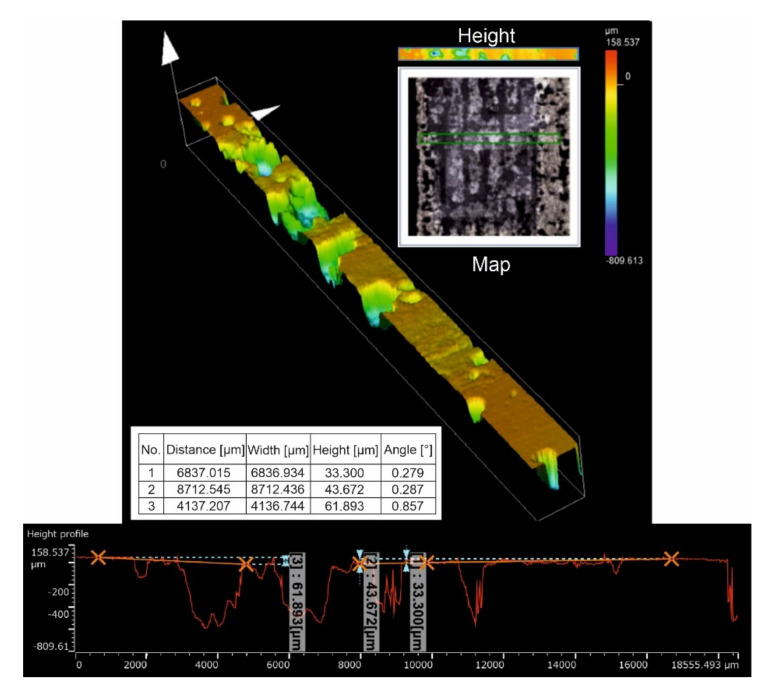
Image and surface profile after the metal-mineral abrasion test of the composite coating (T300 sample) obtained with a scanning laser microscope.

**Figure 20:**
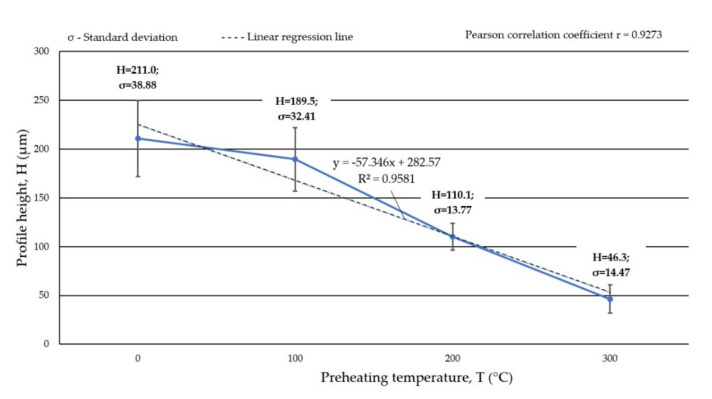
Dependence of the height of the profile of the abrasion area of the composite coating surface on the preheating temperature of the substrate material.

The metal-mineral abrasion resistance of the composite coating was determined by calculating its average volume loss after the ASTM G65 test, Table 6. The results obtained were related to the average share of the base metal dilution in the surface layer, Table 4. The character of the abrasive wear of the top coat was assessed on the basis of visual tests (Figure 18) and observations using a scanning laser microscope (Figure 19), determining the average height of the abrasion area profile depending on the preheating temperature of the base material, Figure 20.

The metal-mineral abrasion test according to ASTM G65, Procedure A, was carried out under medium stress because the sand grains after their interaction with the surface of the test sample were only partially crushed. The mass loss of the composite coating after the abrasive test decreased with increasing preheating temperature of the base material and density of the padding weld. The maximum mass loss of the composite coating, which exceeded 0.08 g, was recorded for the sample made without preheating the base material before padding, and the smallest for the padded coating with preheating the base material to 300 °C. The increase in mass loss is related to the decrease in the density of the composite coating caused by its external and internal porosity, Table 5. The high stress in the layer is also important due to rapid heat dissipation from the unheated substrate, which causes cracks on the surface of the coating and promotes the fragmentation of TiC particles as a result of brittle fracture. Analysis of surface condition after the abrasion test showed an abrasive wear mechanism. The strongly embedded and evenly distributed hard phase in the form of TiC and PCD particles in the matrix of the cobalt alloy constituted a natural and effective barrier for the abrasive medium. 

**Figure 21:**
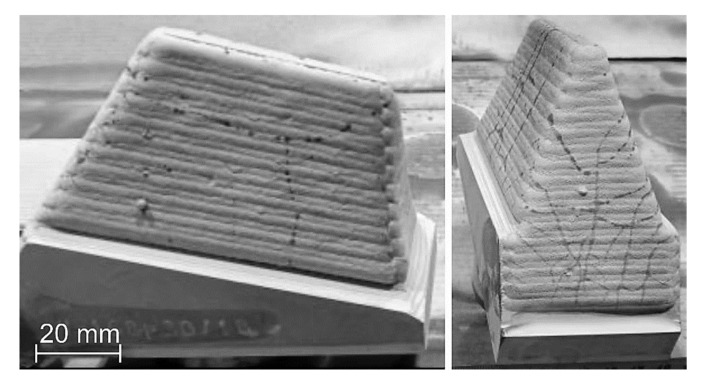
View of the tooth surface of the prototype three-bite auger filled with LDMD with a designed composite filler.

The main wear mechanism of the hardfaced coating was micro-cutting in the form of continuous scratches parallel to the weld axis and, to a much lesser extent, grooving of the surface. The course of the cracks deviated from the rectilinear direction in some places, which indicated the effectiveness of the strengthening of the base material and confirmed the presence of hard phases in the structure. The sand formed scratches on the surface of the composite coating with a profile height ranging from 33 to about 240 µm. The width of the crack profile ranged from 400 as much as over 2000 μm, with the deepest and widest dimensions of the wear profile observed at the overlapping point of successive runs of padding welds. In the area of surface abrasion, single spherical craters were found, remaining after the synthetic PCD particles peeled from the matrix. The wear mechanism noted for microcutting a coating welded by LDMD with a Co-Cr-W-Mo alloy binder was confirmed by Lin et al. [49]. In relation to previously published data [43] and our own research [28] on the wear resistance of layers padded with composite binders in the cobalt alloy matrix with the addition of a hard phase in the form of TiC particles, a beneficial effect on abrasive wear was observed from the addition of synthetic PCD particles. In the case of abrasion-resistant composite layers on a cobalt matrix, the abrasive wear resistance increases with an increase in the volume fraction of the ceramic reinforcement. In the case of the designed alloy, the high resistance to abrasive wear was the result of the type, size and shape in the particles of the hard matrix strengthening phase and the test conditions specified in ASTM G65. In the working conditions of the drilling tool, the influence of natural factors is necessary, which include mechanical load, soil structure, hydrogeological considerations and humidity conditions. These factors were not included in the abrasion resistance assessment of the tested composite coating. Currently, LDMD surfacing technology from the teeth of the prototype three-cone bite (Figure 21) using a designed alloy is being refined. The finished tool will be tested under field conditions. The surfacing layer will be subjected to an assessment of resistance to abrasive, impact and thermal wear.

## 4. Conclusions

The purpose of this research was to assess the effect of the preheating temperature of the base material—low-alloy structural steel of grade AISI 4715—on the susceptibility to cracking, resistance to metal-mineral abrasive wear and metallographic structure LDMD coating, which was innovative in terms of its chemical composition and the type of hard matrix reinforcement phase. In the case analyzed, the substrate material was a low-alloy structural steel AISI 4715 for which the chemical equivalent of carbon CEV = 0.65%. It is assumed that, with CEV values higher than 0.60%, steel is considered difficult to weld, and therefore requires the use of additional treatments regardless of the size and weight of the element being welded. Then, precautions such as preheating the material and maintaining this temperature throughout the surfacing process should be applied. After surfacing, it is also recommended to cool the element very slowly using heating mats, as well as often additional heat treatment. Preheating and meeting the temperature regime during surfacing, especially large components such as a drill bit with milled teeth, are critical factors in terms of surfacing efficiency and weld quality. This treatment allows for an increase in the speed of surfacing, a better melting and flow of the alloy, and a reduction in the likelihood of thermal decomposition of the carbide phase. The results allow us to conclude that the selected chemical composition of the metallic phases as well as the type, amount and size of the hard phase particles of the developed composite powder allow for high accuracy, repeatability of dosing and near perfect melting with the use of laser powder deposition systems. Preheating the base material above 300 °C significantly reduces the susceptibility to cracking and porosity of the metal deposit, reduces internal stresses in the TiC (preventing particle brittleness) and significantly increases resistance to metal-mineral abrasive wear. The process of LDMD with direct feeding of the powder to the weld pool helps to maintain the structural and thermal stability of the synthetic PCD particles. The tungsten from the protective coating of the PCD particles slightly enriches the matrix of the composite and does not significantly increase the hardness measured on the outer surface and the cross section of the surface coating.

## 5. Patents

The procedure for granting a patent (No. P435997) was initiated before the Patent Office of the Republic of Poland.

## Figures and Tables

**Table 1:** Chemical composition of AISI 4715 low-alloy structural steel according to manufacturer data (TimkenSteel Ltd., Canton, OH, USA).

Element, wt.(%)									
Fe	C	Mn	Cr	Mo	Ni	Si	P	S	CEV ^1^
Bal.	0.12–0.18	0.65–0.95	0.40–0.70	0.45–0.60	0.65–1.00	0.15–0.35	≤0.015	≤0.015	0.66

^1^ CEV—Carbon Equivalent Value.

**Table 2:** Chemical composition of powder.

Chemical Composition of the Matrix, wt.(%)									Ceramic Reinforcement of the Matrix, wt.(%)	
Co	Cr	W	Ni	Fe	Mn	Mo	C	Si	TiC	PCD-W
Bal.	24–28	12–14	≤3	<5	≤2	≤1	2.5–3	≤1	90	10

Notes: Carbide to matrix ratio: 60/40 wt.(%).

**Table 3:** Optimum parameters for laser surfacing with Co3+TiC+PCD powder on AISI 4715 steel.

Process Parameters	Value of the Parameter
Laser Power (W)	1800
Scanning Speed (mm/s)	8
Laser Spot Size, (mm)	5
Powder Feed Rate (g/min)	24
Overlap Ratio (%)	33
Heat Input ^1^ (J/mm)	225

^1^ defined as the laser power divided by the scanning speed.

**Table 4:** Results of Rockwell C hardness measurement on the outer surface of the coating laser welded with Co3+TiC+PCD composite powder on AISI 4715 low-alloy structural steel.

Hardnesses, (HRC)							Standard Deviation	Dilution Ratio, (%)
Specimen Number	Measurement Point Number					Average Hardness of the Tested Samples		
	1	2	3	4	5			
T 0	59.8	61.8	60.9	61.7	60.3	60.9	0.9	2.6
T 100	60.4	59.5	61.2	59.2	60.0	60.1	0.8	3.3
T 200	60.2	57.9	59.4	58.7	59.9	59.2	0.9	6.5
T 300	57.4	58.5	59.7	59.0	58.8	58.7	0.8	8.2

**Table 5:** The results of the density measurements together with the calculations of the degree of porosity of the top coat laser-welded with the Co3+TiC+PCD-W composite powder on the AISI 4715 low-alloy structural steel.

Physical Quantity	Average Value of the Measured Quantity for Samples			
	T0	T100	T200	T300
Density ρ (g/cm^3^)	5.7785	6.0176	6.3805	6.6169
Standard Deviation σ_ρ_	0.4092	0.2908	0.2254	0.1978
Open Porosity P_o_ (%)	6.6467	6.0041	2.7647	1.8161
Closed Porosity P_c_ (%)	3.5316	2.0263	0.8925	1.0648
Apparent Density ρ_a_ (g/cm^3^)	5.1903	5.5344	6.1472	6.4263
Total Porosity P_c_ (%)	10.1783	8.0304	3.6572	2.8809

**Table 6:** The results of the metal-mineral abrasive wear resistance test of the composite coating welded with the LDMD method according to ASTM G65.

Specimen Designation	Mass before Test, (g)	Mass after Test, (g)	Mass Loss, (g)	Average Mass Loss, (g)	Clad Layer Density, (g/cm^3^)	Average Volume Loss, (mm^3^)
No preheating						
T 0_1	149.8935	149.8203	0.0732	0.0806	5.7785	13.9482
T 0_2	149.8704	149.7824	0.0880			
Preheating temperature, T = 100 °C						
T 100_1	149.4675	149.3991	0.0684	0.0613	6.0176	10.1867
T 100_2	150.2985	150.2443	0.0542			
Preheating temperature, T = 200 °C						
T 200_1	150.8568	150.8377	0.0191	0.0184	6.3805	2.8837
T 200_2	149.7372	149.7195	0.0177			
Preheating temperature, T = 300 °C						
T 300_1	149.9748	149.9639	0.0109	0.0101	6.6169	1.5263
T 300_2	149.8394	149.8301	0.0093			

## Data Availability

The data are not publicly available due to the initiation of a patent procedure (No. P435997).
